# Removal of Four Inches of the Tibia, for Necrosis

**Published:** 1861

**Authors:** M. M. Eaton

**Affiliations:** Peoria, Ills.


### REMOVAL OF FOUR INCHES OF THE TIBIA, FOR NECROSIS.

By M. M. EATON, M. D., of Peoria.

A Chamberlain, Esq , brought his boy, aged four years, to my office, May 15th, 1861, stating that he lived in Tazewell Co. Ill.. and had been sent to me by Dr. McColl, (his family physician.)

Said that the boy fell from a door-step about one year since, a distance of eight or ten inches, that nothing particular was noticed till about one week afterward, when the knee joint of the left limb began to swell. This extended both up and down the limb, till the whole in three weeks became double its former size. Under treatment, however, this subsided in about four weeks and an abscess formed about an inch above the inner malleolus and considerable offensive pus was discharged.

Soon three other ulcers formed about the middle of the shaft of the Tibia. These also discharged offensive matter, and three small spicula of bone, and gradually merged in one. The discharge has continued till now, all efforts to heal the ulcer having proved abortive.

On examination, I found an ulcer, situated over the shaft of the Tibia, in its upper third, in length about one and a half inches and width one inch, exposing one inch in length of the bone, which was evidently badly necrosed and by probing I found that the disease extended about three inches. The general health of our little patient was good, the whole of the affected limb being, however, considerably atrophied.

I stated the case to the father, and recommended the exsec tion of the diseased bone, to the performance of which he immediately consented. I called in Dr. Hamilton to administer Chloroform, and proceeded at once to the operation. I found the periosteum considerably diseased, but by incising it and using some care, I succeeded in peeling it off with a blunt instrument and leaving it *in situ* in the wound.

Nothing unusual occurred. I removed four inches of bone, so as to be sure and leave only that which was sound, placed a tent in the wound and applied the usual dressings. The ulcer, two days after, began to heal around the edges. The wound healed by healthy granulations from the bottom, and in four weeks both wound and ulcer were entirely healed.

Now, six weeks after the operation, the boy is able to walk well on the limb, new bone a little larger than the original having been deposited. There is left no obvious shortening or deformity.

